# RACIAL DIFFERENCES IN ELEVATED C-REACTIVE PROTEIN AMONG U.S. OLDER ADULTS

**DOI:** 10.1093/geroni/igz038.3018

**Published:** 2019-11-08

**Authors:** Heather R Farmer, Heather R Farmer, Linda A Wray, Hanzhang Xu, Ying Xian, Neha Pagidipati, Eric D Peterson, Matthew E Dupre

**Affiliations:** 1 Duke University, Durham, North Carolina, United States; 2 Pennsylvania State University, University Park, Pennsylvania, United States

## Abstract

C-reactive protein (CRP) is a marker of inflammation linked to numerous acute and chronic conditions. Studies have not considered racial differences in elevated CRP among older adults at the national level. We investigate racial differences in elevated CRP and the socioeconomic, psychosocial, behavioral, and physiological factors that contribute to these differences overall and by gender using a nationally-representative prospective cohort of 14,700 non-Hispanic black and white participants in the Health and Retirement Study followed from 2006 to 2014. Random effects logistic regression models showed that blacks were more likely to have elevated levels of CRP than whites. In men, the racial differences in elevated CRP were attributed to a combination of socioeconomic, psychosocial, and behavioral factors. In women, the racial differences in elevated CRP were primarily attributable to physiological factors. The findings from this work have potentially important implications for clinical practice and interventions targeting vulnerable segments of the population.

